# Multi-Objective Human Resource Allocation Approach for Sustainable Traffic Management

**DOI:** 10.3390/ijerph17072470

**Published:** 2020-04-04

**Authors:** Soumendra Nath Sanyal, Izabela Nielsen, Subrata Saha

**Affiliations:** Department of Materials and Production, Aalborg University, Fibigerstrede 16, DK 9220 Aalborg, Denmark; soumen.sanyal0@gmail.com (S.N.S.); izabela@mp.aau.dk (I.N.)

**Keywords:** manpower allocation, binary programming, fuzzy-efficient solution, emission control

## Abstract

Efficient human resource deployment is one of the key aspects of road traffic management for maintaining the lifelines of any metropolitan city. The problem becomes relevant when collaboration between human resources with different skills in day-to-day operations is necessary to maintain public and commercial transport, manage various social events and emergency situations, and hence reduce congestion, injuries, emissions, etc. This study proposes a two-phase fuzzy multi-objective binary programming model for optimal allocation of five different categories of human resources to minimize the overall operational cost, maximize the allocation to accident-prone road segments, minimize the number of volunteer personnel and maximize the direct contact to reduce emissions and road traffic violations, simultaneously. A binary programming model is formulated to provide an efficient individual manpower allocation schedule for multiple road segments at different shifts. A case study is proposed for model evaluation and to derive managerial implications. The proposed model can be used to draw insights into human resource allocation planning in traffic management to reduce road traffic congestion, injuries and vehicular emissions.

## 1. Introduction

Efficient traffic management is a major concern for most highly populated cities worldwide, due to the exponential growth of transport and movement of rural population, causing prolonged Road Traffic Congestion (RTC), Road Traffic Injuries (RTI), vehicular emissions, etc. [1]. Diakaki et al. [2] stated that RTC has become a major threat to the socio-economic structure of modern societies. In the year 2015, the World Health Organization (WHO) reported that 90 percent of RTI occur in low-middle income countries and are responsible for many deaths and life-long disabilities [3]. RTI is a leading death factor, causing 1.35 million deaths each year around the world [4]. According to the Institute for Health Metrics and Evaluation (IHME), RTI were responsible for approximately 907,900, 1.3 million and 1.4 million annual causalities around the world in the years 1990, 2010 and 2013 respectively [5]. To improve operational efficiency, several measures have been initiated such as restructuring the road network, integrating modern technologies to control road traffic, providing pedestrian facilities in accident-prone areas and organizing social campaigns to increase awareness for the common people ([6,7]). In highly populated countries like India, traffic management still remains a major concern because RTI are the sixth leading cause of death in India with a greater share of hospitalization, deaths, disabilities and socio-economic losses [8]. RTI causes a huge burden on the health sector of India, in terms of pre-hospitalization, acute care and rehabilitation [9]. In a recent annual report on road accidents in India the following alarming results were reported [10]:

- Number of road accidents in India was 464,910 in 2017, causing 147,913 deaths and 470,975 injuries
- The top eleven Indian cities, namely Bhopal, Chennai, Delhi, Hyderabad, Indore, Jabalpur, Jaipur, Kochi, Kolkata, Mumbai, and Mallapuram accounted for 51.1% of the RTI

Proper allocation of manpower is one of the crucial factors for ensuring road safety. In this research, we formulated a multi-objective linear programming (MOLP) model to obtain an efficient manpower allocation schedule. The objective was to find a compromise solution by considering four objectives as follows: (1) total operational cost minimization, (2) maximization of allocation of traffic sergeants to cover accident-prone road segments, (3) minimization of number of volunteer personnel to be used, and (4) maximization of direct contact to reduce vehicular emissions and road traffic rule violations. The proposed model considered various road segments, duty-shifts in a day, strength of different types of traffic police resources, road accident details, traffic rule violations etc. A two-phase approach was used to find a Pareto-optimal solution that ensured a trade-off between government expenditure and road traffic safety-related issues.

Road Traffic management has become an urgent problem in India [11]. In a recent report by Financial Express [12], it is highlighted that traffic congestion is a major problem in Kolkata, India. In this study, a mathematical model is formulated to explore the characteristics of such issues. Kolkata is considered to be one of the densely populated cities with many slow and medium-speed moving vehicles like hand-pulled rickshaws, auto rickshaws, public buses and trams along with the exponentially increasing number of high speed vehicles. Lack of public awareness makes the road traffic flow worse during peak hours and pedestrians frequently use main roads instead of pedestrian paths, resulting in fatal injuries and death cases on a regular basis. Maintaining the flow of these large numbers of vehicles and provide safety for pedestrians is a challenging issue for Kolkata Traffic Police (KTP). In addition, political gatherings, processions and cultural carnivals take place on a regular basis in almost every part of the city. KTP is also responsible for managing all these events and ensuring a smooth traffic flow. Traffic law or emission rule violations frequently take place on roadways. The government continuously makes efforts to mobilize road traffic by integrating electronic devices such as CCTV, speed limit controllers, etc., and organizing social awareness programs like “Safe Drive Save Life (SDSL)” on a regular basis. The main objective of SDSL is to educate the public about fundamental traffic rules and regulations. Recently, the government hired a large number of Civic-Volunteers and Home-Guards to reduce accidents and casualties. The main job of volunteers of KTP is to support respective units for traffic management as well as control the road segments during major festivals, meetings, rallies, and demonstrations, etc. Volunteers are also engaged in official work such as traffic fine collection, managing unauthorized parking of vehicles, vehicular emission control, creating pedestrian awareness, etc. [6,13]. In this study, a mathematical model is proposed to highlight the characteristics of traffic manpower allocation schemes and explore insights from the perspective of obtaining a decision support scheme for traffic manpower management systems.

### Literature Review

RTI have become a major problem in highly populated countries like India, China, Thailand and Malaysia as well as in several parts of Africa ([3,14,15,16,17]). Lee et al. [18] proposed a complete analytical model for the allocation of patrolmen to highways. The authors formulated an integer goal-programming model by considering highway road segments and shifts to find optimal allocations. Modelling aspects of urban patrol manpower allocation problems were investigated by Kwak and Leavitt [19] by considering different social issues. The authors integrated scenarios such as prevention of motorway crimes, the establishment of peace and harmony of commuters on roadways in emergency situations when developing the model. Basu and Ghosh [20] proposed a multi-objective model for the allocation of police patrolmen in metropolitan cities. The authors employed non-linear goal programming to formulate the model. D’Amico et al. [21] employed simulated annealing search techniques for police command. The authors formulated a Patrol Car Allocation Model (PCAM) for the allocation of police resources. Balaji and Srinivasan [22] proposed a multi-agent traffic management system based on type-2 fuzzy logic. The authors compared the performance of proposed multi-agent controller with a hybrid neural network based multi-agent system and real-time adaptive traffic controller. Zhao et al. [23] presented a minimax programming model for the optimal dispatch of traffic and patrol police service platforms. A location allocation model for traffic police patrol vehicles in an interurban network was proposed by Adler et al. [24]. The authors investigated the assignment problem of traffic police patrol route planning. He et al. [25] proposed the concept of shortest path evacuation routing with traffic police resource allocation in city transportation networks. Parr and Wolshon [26] proposed a manual traffic control model to handle emergency situations and planned special events. The study undertook four primary tasks: the collection and processing of video footage of police officers, the development of a discrete choice model, programming the proposed model into a microscopic traffic simulator and validating the model. In a recent study by Adler et al. [27], it was reported that the influence of traffic police is significant for road safety in the context of northern Israel. Dunnett et al. [28] developed an optimization system for police dispatch to manage incidents occurring in real time. Gudwin et al. [29] proposed a multi-purpose enhanced cognitive architecture for urban traffic controllers. A traffic management simulator was developed to test the junction manager with four different topologies. Differing from the existing literature, we formulated a binary programming model in a multi-objective formulation to obtain insights in the allocation of five different manpower resources on the government expenditure, safety and emission reduction goals.

In multi-objective programming problems, it is always challenging to attain the ideal solution due to the conflicting nature of objective functions. Researchers have proposed several methods such as weighted sum [30], goal programming [31], global criterion [32], reference point [33], the step method [34], epsilon-constraints [35] and others. An overview of recent developments in this stream of research can be found in Ben-Tal et al. [36]; Tanino et al. [37]; Shapiro et al. [38] and others. Consequently, the issue of selecting a particular method in a given context is still a subject of active research and largely depends on the decision makers. However, after the pioneering study by Zadeh [39], fuzzy set theory has become an efficient methodology for solving multi-objective programming problems (Zimmermann [40]; Sakawa et al. [41]; Moghaddam [42]; Moon et al. [43]; Chung et al. [44]). Although the max-min approach [40] is frequently employed to solve multi-objective programming problems due to computational simplicity, the method does not always ensure a non-dominated fuzzy-efficient solution [45]. In order to overcome this problem, researchers devised a two-phase approach (Guua and Wu [46]; Arikan and Güngör [47]). In the two-phase approach, the decision maker can achieve the highest membership degree of the objectives and a fuzzy-efficient solution. However, Jimenez and Bilbao [48] proved that a fuzzy-efficient solution might not always be a Pareto-optimal solution. Therefore, Wu et al. [49] recently modified the method of Jimenez and Bilbao [48] for ensuring the Pareto-optimal solution by redefining membership functions in fuzzy environments. The authors proved that the solution methodology guarantees a Pareto-optimal solution with additional robust information for decision makers and is easy to implement. Consequently, we used two-phase method to find the allocation scheme. In addition, the method was used for solving production planning problem [43]; closed-loop supply chain network design problem [50]; and an optimal food preparation problem [51], etc.

## 2. Mathematical Model

In this research, a MOLP model is proposed to achieve an efficient traffic personnel allocation scheme to deal with important scenarios related to road traffic management such as RTI, RTC, response to emergency situations, traffic rules violations, etc. We considered the allocation of five different types of human resources, namely Assistant sub-inspectors (a), Sergeants (s), Constables (c), Home-Guards (h) and Civic-Volunteers (v). Of the first three types of personnel, Assistant sub-inspectors are responsible for directly monitoring incidents on roads; Sergeants are mainly responsible for patrolling and Constables are responsible for surveillance through electronic devices in office and legal aspects of the traffic department, and are directly associated with the traffic department. However, Home-Guards and Civic-Volunteers are involved in the system as casual employees [6]. Therefore, the first three types are resource constrained, but Government can hire Home-Guards and Civic-Volunteers according to its needs. To formulate the problem, the following notations are used throughout the article.
List of notations
Sets
Ttraffic manpower resources types (index: t); t = a, s, c, h, v$N_{t}$number of available manpower resources of types *t*, ∀t*i*number of road segments, *i* = 1, 2, … , m*j*number of shifts, *j* = 1, 2, … , n*s*special event of event types, *s* = 1, 2, … , pParameters
$E_{ij}$minimum number of total traffic personnel that need to be allocated to *i*-th road-segment and *j*-th shift$Cost^{t}$operational cost of traffic personnel of type *t*$Ln_{i}$length of *i*-th road$p_{ij}$$p_{ij}=1$ if there is possibility a special event occurring or higher traffic flow in *i*-th road-segment and *j*-th shift; $p_{ij}\;=\;0$, other wise$RV^{t}$average number of cases logged by traffic personnel of type *t* in each shift$SE_{sj}$special event of type *s* occurred at *j*-th shift, $SE_{sj}\in \left\{0,1\right\}$$M_{s}$minimum number of personnel that needs to be allotted to *s*-th event$SR_{j}$minimum number of personnel that needs to be allotted to surveillance at *j*-th shift$EM_{j}$minimum number of personnel that needs to be allotted to emission controlsDecision Variables
$x_{ijk_{t}}^{t}$$x_{ijk_{t}}^{t}=1$, if $k_{t}$-th traffic personnel of type *t* is allocated to *i*-th road segment in *j*-th shift; $x_{ijk_{t}}^{t}=0$, otherwise

Based on the above-mentioned notations, the following multi-objective binary manpower scheduling problem is formulated:

- The first objective minimizes the sum of total allocation cost in all roads and shifts in a particular day:
  $$\mathrm{Min}\;G_{1}=\sum_{i=1}^{m}\sum_{j=1}^{n}\sum_{k_{a}=1}^{N_{a}}Cost^{a}x_{ijk_{a}}^{a}+\sum_{i=1}^{m}\sum_{j=1}^{n}\sum_{k_{s}=1}^{N_{s}}Cost^{s}x_{ijk_{s}}^{s}+\sum_{i=1}^{m}\sum_{j=1}^{n}\sum_{k_{c}=1}^{N_{c}}Cost^{c}x_{ijk_{c}}^{c}$$
  (G1) $$+\sum_{i=1}^{m}\sum_{j=1}^{n}\sum_{k_{h}=1}^{N_{h}}Cost^{h}x_{ijk_{h}}^{h}+\sum_{i=1}^{m}\sum_{j=1}^{n}\sum_{k_{v}=1}^{N_{v}}Cost^{v}x_{ijk_{v}}^{v}\;$$
  Therefore, G1 ensures that the overall allocation cost remains manageable.
- The second objective maximizes the allocation of Sergeants to all accident-prone roads:
  (G2) $$\mathrm{Max}\;G_{2}=\sum_{i=1}^{m}\sum_{j=1}^{4}\sum_{k_{s}=1}^{N_{s}}\frac{p_{ij}}{Ln_{i}}x_{ijk_{s}}^{s}\;$$
  Therefore, based on the prior information, more Sergeants need to be allocated on accident-prone road segments.
- The third objective minimizes the total number of volunteer personnel in the entire allocation scheme:
  (G3) $$\mathrm{Min}\;G_{3}=\sum_{i=1}^{m}\sum_{j=1}^{n}\sum_{k_{h}=1}^{N_{h}}x_{ijk_{h}}^{h}+\sum_{i=1}^{m}\sum_{j=1}^{n}\sum_{k_{v}=1}^{N_{v}}x_{ijk_{v}}^{v}\;$$
  Therefore, G3 ensures that manpower in the existing system should be utilized as efficiently as possible.
- Finally, the fourth objective maximizes the number of direct contracts to identify traffic and emission rule violations:
  $$\mathrm{Max}\;G_{4}=\sum_{i=1}^{m}\sum_{j=1}^{n}\sum_{k_{a}=1}^{N_{a}}RV^{a}x_{ijk_{a}}^{a}+\sum_{i=1}^{m}\sum_{j=1}^{n}\sum_{k_{s}=1}^{N_{s}}RV^{s}x_{ijk_{s}}^{s}+\sum_{i=1}^{m}\sum_{j=1}^{n}\sum_{k_{c}=1}^{N_{c}}RV^{c}x_{ijk_{c}}^{c}$$
  (G4) $$+\sum_{i=1}^{m}\sum_{j=1}^{n}\sum_{k_{h}=1}^{N_{h}}RV^{h}x_{ijk_{h}}^{h}+\sum_{i=1}^{m}\sum_{j=1}^{n}\sum_{k_{v}=1}^{N_{v}}RV^{v}x_{ijk_{v}}^{v}\;$$
  Note that G4 maximizes the direct contract related with rule violation; emission etc. Traffic rule violations and vehicular emissions have become regular occurrences in India. In particular, the city Kolkata is considered to be one of the most polluted cities in India in terms of air pollution. Vehicular emission is considered to be one of the key causes of this poor situation. On the other hand the number of vehicles are increasing rapidly and the violation of traffic rules has become a common phenomenon. KTP Yearbook stated that 7,470,380 cases were lodged against traffic rule violation and 102,943 cases were lodged against rash driving in 2017. KTP is not only committed to maintaining smooth traffic flow but also to managing these rule violations. KTP employs dedicated teams to control these situations. Therefore, we incorporate this goal so that direct contact can be improved as much as possible [6,13]. The functional and operational constraints for the proposed multi-objective optimization model are as follows:
  (1) $$\sum_{j=1}^{4}x_{ijk_{t}}^{t}\leq 2\;\forall \;i,\;k_{t},\;t=a,s,c,h,v$$
  (2) $$\sum_{k_{a}=1}^{N_{a}}x_{ijk_{a}}^{a}+\sum_{k_{s}=1}^{N_{s}}x_{ijk_{s}}^{s}+\sum_{k_{c}=1}^{N_{c}}x_{ijk_{c}}^{c}+\sum_{k_{h}=1}^{N_{h}}x_{ijk_{h}}^{h}+\sum_{k_{v}=1}^{N_{v}}x_{ijk_{v}}^{v}\geq E_{ij}\;i\in I,j\in J,k_{t}\in N_{t}$$
  (3) $$\sum_{k_{t}=1}^{N_{t}}x_{ijk_{t}}^{t}\leq M.x_{ijk_{c}}^{c}\;\forall \;i,j,k_{t},t=h,v$$
  (4) $$\sum_{k_{t}=1}^{N_{t}}x_{ijk_{t}}^{t}\leq M.x_{ijk_{a}}^{a}\;\forall \;i,j,k_{t},t=h,v$$
  (5) $$x_{ijk_{t}}=0\;i\in I,j\in J,k_{t}\in N_{t},t=a,s,c$$
  (6) $$\;x_{ijk_{t}}^{t}+x_{i\left(j+1\right)k_{t}}^{t}\leq 1,\;\forall \;i,j,k_{t}$$
  (7) $$\sum_{k_{a}=1}^{N_{a}}x_{ijk_{a}}^{a}+\sum_{k_{s}=1}^{N_{s}}x_{ijk_{s}}^{s}+\sum_{k_{c}=1}^{N_{c}}x_{ijk_{c}}^{c}+\sum_{k_{h}=1}^{N_{h}}x_{ijk_{h}}^{h}+\sum_{k_{v}=1}^{N_{v}}x_{ijk_{v}}^{v}\geq SE_{sj}M_{s}\;i\in I,j\in J\;\;$$
  (8) $$\sum_{k_{a}=1}^{N_{a}}x_{ijk_{a}}^{a}+\sum_{k_{s}=1}^{N_{s}}x_{ijk_{s}}^{s}\geq SR_{j}\;\forall \;i,j$$
  (9) $$\sum_{k_{v}=1}^{N_{v}}x_{ijk_{v}}^{v}\geq EM_{j}\;\forall \;i,j$$
  (10) $$\;x_{ijk_{t}}^{t}\in \left\{0,1\right\}$$
  Constraint (1) ensures that an individual traffic personnel cannot be allocated to more then two shifts in a day. Constraint (2) represents the minimum requirement of traffic resources in jth shift and ith road segment. This constraint ensures that at least some manpower should be allocated to each road and shift from all five types of personnel. Constraint (3) and (4) represent the allocation of volunteer traffic resources, i.e., Home-Guards (h), and Civic- Volunteers (v) can be assigned with either an ASI or a Constable. Constraint (5) ensures the unavailability of ASIs, Sergeants, and Constables on a particular day. Constraint (6) represents that the allocation of ASIs, Sergeants and Constables is not possible for two consecutive shifts. Note that Home-Guards or Civic-Volunteers can be allocated in consecutive shifts. Constraint (7) represents the allocation of tth type traffic resources to manage sth type of special events occur in jth shift. Constraint (8) represents the allocation of ASIs and Sergeants for surveillance purposes in ith road segment and jth shift. Constraint (9) represents the allocation of Civic-Volunteers for emission reduction purposes in jth shift. The complexity of the above multi-objective binary linear programming model is a function of problem size affected by the set of road segments, set of shifts, number of five types of manpower resources, and therefore the model consists of $IJN_{a}N_{s}N_{c}N_{h}N_{v}$ binary decision variables.

## 3. Solution Procedure

In order to explain how to use the two-phase method to obtain a fuzzy-efficient solution for the MOLP model, the following definitions are used:

**Definition** **1.** *A multiple objective optimization problem can be represented as follows:*

$$\left\{\begin{array}{ll} opt & \left(f_{1}\left(x\right),f_{2}\left(x\right),\ldots ,f_{k}\left(x\right)\right)\\ s.t. & x\in X=\{x|g_{j}\left(x\right)\leq 0,j=1,\cdots ,m\} \end{array}\right.$$

*where “opt” denotes minimization or maximization;*$x=(x_{1},x_{2},\ldots ,x_{n})$*are the decision variables;*$f_{i}\left(x\right),\left(i=1,\ldots ,k\right)$*are multiple objective functions to be optimized;*$X\subset R^{n}$*satisfies the set of system constraints*$g_{j}\left(x\right)\leq 0,(j=1,\leq \cdots ,m$*)* [43].

**Definition** **2.** *A decision plan*$x^{0}\in X$*is said to be a Pareto-optimal solution to the multiple objective optimization problem if no other*y∈X*, exists, such that*$f_{k}\left(y\right)\leq f_{k}\left(x^{0}\right)$*for all k and*$f_{s}\left(y\right)<f_{s}\left(x^{0}\right)$*for at least one s (Wu et al.* [49]*).*

**Definition** **3.** *A decision plan*$x^{0}\in X$*is said to be a fuzzy-efficient solution to the model if no other*y∈X*exists, such that*$\mu_{k}\left(f_{k}\left(y\right)\right)\geq \mu_{k}\left(f_{k}\left(x^{0}\right)\right)$*for all k and*$\mu_{k}\left(f_{s}\left(y\right)\right)>\mu_{s}\left(f_{s}\left(x^{0}\right)\right)$*for at least one s (Wu et al.* [49]*).*

For MOLP model, optimal values for all the objectives cannot be achieved simultaneously and researchers are seeking Pareto-optimal solution, which prevents the improved solution for an individual objective from worsening one or more other objectives. However, Pareto-optimally does not ensure a fuzzy-efficiency solution ([48]). Therefore, we employed the two-phase method ([49]) to obtain a solution for the proposed MOLP model. To solve the proposed multi-objective human resource scheduling problem, we employed the following steps [49]:

Step 1: Determine the positive ideal solution(${G_{r}}^{min}$) and the negative ideal solution(${G_{r}}^{max}$) for each objective function (r=1,2,3,4) by solving each objective function while ignoring the other objective function subject to set of constraints.
Step 2: Construct linear membership functions featuring both the continuously increasing property of the maximization objective function and the decreasing property of the minimization objective function. For the maximization objective goal (r=1,2,4):
  $$\mu_{max}\left(G_{r}\right)=\left\{\begin{array}{cc} \frac{G_{r}-{G_{r}}^{min}}{{G_{r}}^{max}-{G_{r}}^{min}} & \mathrm{if}\;{G_{r}}^{min}\leq G_{r}\leq {G_{r}}^{max}\\ 0 & \mathrm{if}\;G_{r}\leq {G_{r}}^{min} \end{array}\right.$$
  For the minimization type objective function (r=3):
  $$\mu_{min}\left(G_{r}\right)=\left\{\begin{array}{cc} \frac{{G_{r}}^{max}-G_{r}}{{G_{r}}^{max}-{G_{r}}^{min}} & \mathrm{if}\;{G_{r}}^{min}\leq G_{r}\leq {G_{r}}^{max}\\ 0 & \mathrm{if}\;G_{r}\leq {G_{r}}^{max} \end{array}\right.$$
  where the possible range for the *r*-th objective is $\left[{G_{i}}^{min},{G_{i}}^{max}\right]$, (r=1,2,3,4).
Step 3: Solve the following optimization problem in Phase 1 as given below:
  Maxλ
  $$s.t.\;\mu_{max}\left(G_{r}\right)\geq \lambda ,\;\mu_{min}\left(G_{r}\right)\geq \lambda ,\forall r,\;\lambda \geq 0\;\mathrm{and}\;\mathrm{set}\;\mathrm{of}\;\mathrm{constraints}\;\mathrm{defined}\;\mathrm{previously}.$$
  Note that membership functions in the two-phase approach do not have an upper or lower bound unlike the conventional max-min operator approach. Therefore, in Phase 1, each objective function might not attain the lowest or highest possible compromise value because in a decision making context, the fuzzy goals are set by the decision maker through subjective domain knowledge. Therefore, if we optimize λ in Phase I, we first obtain a trade-off among all objectives.
Step 4: Finally, Phase II provides the flexibility to reach optimal for each objective by relaxing this constraint. The decision-maker needs to solve the following optimization problem in this regard:
  $$\mathrm{Max}\;\rho_{o1}+\rho_{o2}+\rho_{o3}+\rho_{o4}$$
  $$s.t.\;\mu_{max}\left(G_{r}\right)-\rho_{or}\geq \lambda^{*},\;\forall r=2,4,\;\mu_{min}\left(G_{r}\right)-\rho_{or}\geq \lambda^{*},\forall r=1,3$$
  $$\rho_{or}\geq 0\;\forall r\;\mathrm{and}\;\mathrm{set}\;\mathrm{of}\;\mathrm{constraints}\;\mathrm{defined}\;\mathrm{previously}.$$
  where $\lambda^{*}$ is the optimal value of λ obtained in Phase I. If $\rho_{or}=0$, ∀r, then there are no better efficient solutions for the model compared to Phase I and if $\rho_{or}>0$ for at least one *r*, the solution obtained in Phase II is more efficient. In this way in Phase II, the decision makers have the opportunity to improve the solution based on the each objective. In addition, by comparing, the optimal values of $\rho_{or}$, the decision maker can gain knowledge about the improvement of the previous solution. Two-phase approach has been widely used in recent years due to its flexibility ([52]). Note that the optimal allocation in Phase II is a Pareto-optimal solution ([49]).

In addition, we employed weighted sum method to compare effectiveness of the two-phase solution approach. Note that weighted sum method is also used extensively to solve multi-objective optimization problems ([53,54]). In the weighted sum method, a multi-objective optimization problem of the form

$$\left\{\begin{array}{cc} min & \left(f_{1}\left(x\right),f_{2}\left(x\right),\ldots ,f_{k}\left(x\right)\right)\\ s.t. & x\in X=\{x|g_{j}\left(x\right)\leq 0,j=1,\cdots ,m\} \end{array}\right.$$

the decision maker needs to solve the following optimization problem to obtain solution of the original problem

$$Min\;\sum_{i=1}^{k}w_{i}f_{i},\;\mathrm{subject}\;\mathrm{to},\;\sum_{i=1}^{k}w_{i}=1,\;0\leq w_{i}\leq 1,\;g_{j}\left(x\right)\leq 0.$$

Therefore, $w_{i}$ represents weight factor associated with the ith objective function.

## 4. Case Study

**Figure 1:**
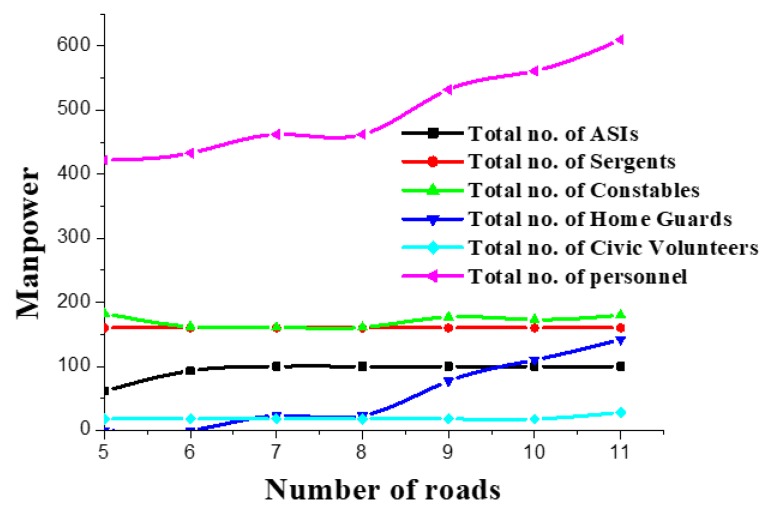
Nature of allocation of manpower with increasing number of road segments.

According to information of Kolkata Traffic Police annual review bulletin [6,13], the range of parameters were estimated. We considered four duty shifts for the traffic personnel in the time interval between 7.00 a.m. to 22.00 p.m. We assumed the availability of traffic personnel to be: a=50, s=80, c=100, h=150, and v=200; respectively. We considered five different types of special events, namely political or social demonstration, religious roadshow or procession, social or political rally, roadblock, and fatal accident. The parameters used to generate numerical instances based on the increasing number of road segments or manpower resources were randomly generated integers or floats from uniform distributions (U(a1;a2)), where a1 and a2 represent a minimum and a maximum value, respectively. For example, the manpower requirements ($M_{s}$) for special events were randomly generated in U(4,10). Similarly, the length of each road segment was signified as ($Ln_{i}$) as U(3,8) in kilometers. The minimum requirement of traffic personnel ($E_{ij}$) in each road and each shift is U(8,15). During each shift, number of Civic-Volunteers ($EM_{j}$) to be allocated for vehicular emission control were U(4,8). Operational costs for allotting a, s, c, h and v were considered as 1200,1500,1000,700,500, respectively. The corresponding average number of cases lodged by each type of manpower were 3,5,3,1,0.5, respectively. We performed a series of numerical experiments of different sizes to obtain insights to human resource allocation with CPLEX. Volunteers from the KTP department would support respective units for traffic management as well as control the road segments during major festivals, meetings, rallies, demonstrations, etc. In addition to this, volunteers were engaged in official work such as traffic fine collection and managing unauthorized parking of vehicles. Therefore, the main objective of KTP is to hire volunteers to ensure people’s safety and handle a large amount of road traffic (see Appendix A). For performance evaluation, all numerical experiments were executed with Intel Core i5-4590 CPU with 3.30 GHz processors and 8.0 GB RAM. The allocation scheme with an increasing number of roads is presented in Figure 1.

From Figure 1, one can see that the overall allocation of human resources increased with number of road segments. We refer to Table A1 in Appendix B for the details of the step-wise solution for Phases 1 and 2. ASIs and Constables were under-utilized, it is necessary to hire Civic-Volunteers for their functionalities, i.e., vehicular emission control. This gives an indication that if proper monitoring and training programs are organized for existing resources then a better allocation schedule can be made without hiring external resources. Therefore, top level management needs to monitor the existing resources. It was found that Sergeants need to be utilized to a higher extent. Although hiring a Home-Guard is more expensive than hiring a Civic-Volunteer, until total number of hire is more due to their working efficiency. By comparing objective values, one can find that a multi objective optimization framework for traffic manpower allocation can provide a robust overview compared to single objective optimization framework. Observing the values of Obj 1 and Obj 4 in Table A1 in Appendix B, one can notice that the Pareto-optimal solution reflects the reality. It is sensible that higher number of manpower allocation in road segment can be expected to reduce number of traffic and emission rule violation, but it can lead to higher cost for Government. Therefore, a trade-off is necessary. For example, the government could reduce operational costs by 49% by compromising 22% of traffic violations, but this could lead to higher costs for the government. We refer to Table A2 in Appendix B for the the allocation obtained using the weighted sum method. To obtain the allocation scheme, we used equal weights for all the objectives. One can observe that the method fails to deliver Pareto-optimal solution for all the instances. Due to their higher cost, no Sergeants are allocated for the first two scenarios. Therefore, the allocation scheme compromised the accident-reduction. Moreover, the highlighted diagonal values in Table A1 in Appendix B representing the optimal solution for individual objectives also demonstrate that the two-phase approach outperforms the weighted sum method.

**Figure 2:**
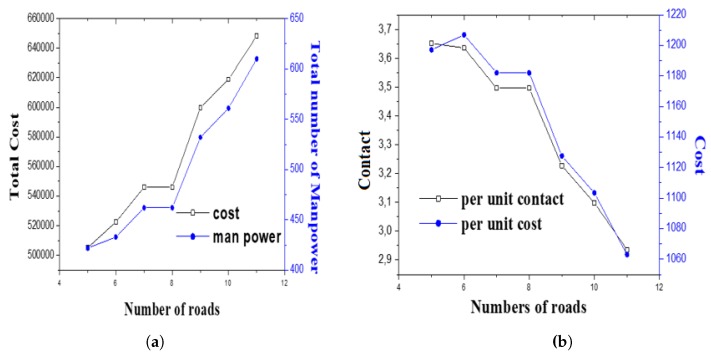
(**a**) Total cost vs. total manpower (**b**) Average rule violation vs. average allocation cost.

Graphical representation of total operational cost, average number of emission and traffic rule violation cases identified by each resource is presented in Figure 2.

One can find that with an increasing number of road segments, the total number of manpower and cost increased (Figure 2a). However, the average cost per allocation and per unit rule violation cases decreased. Because, hiring Home-Guards and Civic-Volunteers is less expensive compared to other available manpower resources. Figure 1 provides an indication that the number of volunteers increased with the number of road segments. Therefore, the results are sensible. However, per unit rule violation identification was also reduced considerably. According to constraint structures for considering the day-to-day operations, some man-power needs to be engaged in handling special events, until per unit contact rule violation decreased significantly. Therefore, government organizations need to find a trade-off before involving too many volunteers in day-to-day operations.

In the formulation and practice, Sergeants are signified as the most important human resource and need to be deployed more on the accident-prone roads. Therefore, we conducted a sensitivity analysis to explore the impact of Sergeants.

**Figure 3:**
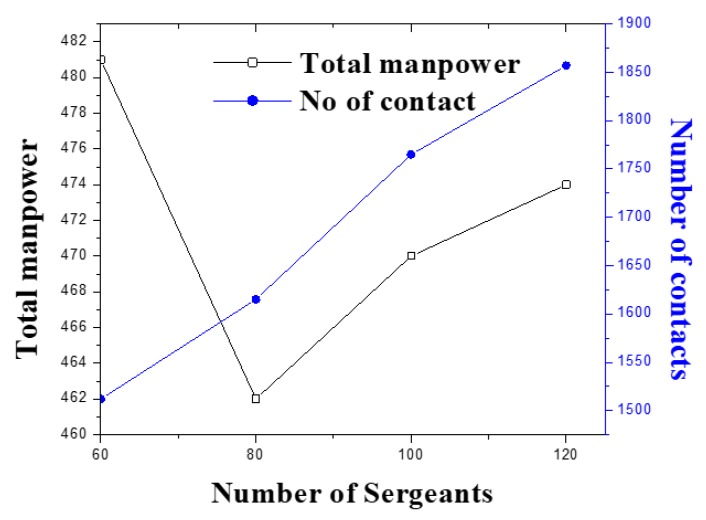
Number of contacts vs. total manpower.

We refer to Table A3 in Appendix B for a detailed overview of solution procedure. Figure 3 shows that it is possible to increase number of traffics and emission rule violations without employing too many additional personnel. In all scenarios, if one optimizes (G1), then the number of Home-Guard increased significantly, however the total number of rule violations is too few. Therefore, a trade-off between involving efficient manpower and operational cost needs to be evaluated before implementing a day-to day schedule. In all scenarios, Sergeants are allocated with their highest resource level in the final solution. This justifies the efficiency of the proposed method.

Finally, we conducted a sensitivity analysis based on the cost of hiring volunteer resources. We refer to Table A4 in Appendix B for the details.

**Figure 4:**
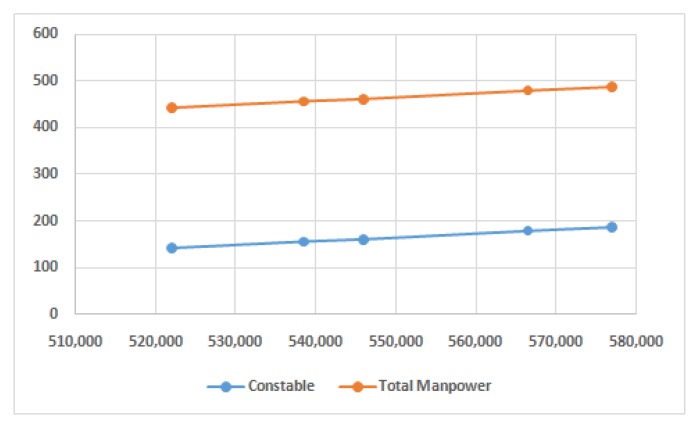
Number of Constables and total manpower allocation with increasing cost of hiring.

Figure 4 justifies that if the cost of hiring external resources increases are increased then it is favorable to utilize the existing resources more efficiently. The numerical results also justify the fact. In all scenarios, 23 Home-Guards and 18 Civic- Volunteers were used. However, the number of Constable allocation also increased. If we keep in mind about the effectiveness of Constable in the fourth objective, then one can find the justification for utilizing more Constable in the day-to-day allocation schedule. Interestingly, the allocation schedule can also increase the number of rule violations. Additionally, there is no need to sacrifice the second objective, remain uniform, which is important in the context of traffic management.

The intensity of vehicular pollution in the city of Kolkata and its periphery has become a major problem as a result of significantly increasing rates of motorized vehicle usage. The abundance of poorly maintained vehicles, use of petrol fuel, and poor controls are responsible for this alarming situation [55]. West Bengal Pollution Control Board reported that the automobiles contribute significantly to particles of size of 1.1 micron and responsible for nearly 50 percent of the air pollution in Kolkata [56]. The government conducted several awareness programs to control the situation but it is necessary to examine the condition of roadside vehicles. The proposed model demonstrates that a trade-off among operational cost, accident, vehicular emission and rule violation reduction goals and the allocation of special types of manpower resources to accident-prone road segments can lead to a sustainable and economical allocation schedule.

## 5. Conclusions

The efficient allocation of human resources in road traffic management has become a challenging issue for densely populated countries like India, Bangladesh, China, Thailand, Malaysia as well as in several parts of Africa. Fatal injuries and causalities have also become a common phenomenon. In this research, we proposed a multi-objective two-step binary programming model to obtain a day-to-day manpower allocation schedule that minimizes overall expenditure, maximizes traffic personnel allocation in accident-prone road segments, minimizes the hiring number of traffic volunteers and maximizes direct contact to reduce traffic rule violations and vehicular emissions. A two-phase approach was used to obtain an allocation scheme for each manpower resource and results were compared with the weighted sum method. Finally, a case study was conducted in the context of manpower allocation related to traffic management.

The results demonstrate that manpower scheduling problems need to be formulated within a multi-objective framework, otherwise the allocation schedule can lead to higher operational cost without satisfying the traffic rule violation and emission control objective or lower cost without too much resource allocation on accident-prone roads. Additionally, a multi-objective framework can lead to maximum utilization of existing resources. The Sensitivity analysis with respect to Sergeants reflected that fulfillment of the second objective can be improved significantly without too great an increase in total allocation costs. The allocation scheme can be used by government organizations such as the Kolkata traffic police, where a trade-off between road safety related issues and operational cost is important. The results reveal that the government needs to monitor existing resources properly before hiring external manpower resources or organize training programs regularly to improve flexibility among existing resources. Through the proper allocation of manpower, traffic management organizations can reduce the occurrence of RTI.

Solution approaches for multi-objective linear optimization methods are not limited and each method has some advantages and drawbacks. Therefore, it is necessary to verify the trends of the solutions with other methods. Regarding the problem formulation, one can modify objective 4 where the number of direct contacts does not increase linearly with the amount of manpower. Due to the growing number of binary variables, we found that computational time increased exponentially. Therefore, as a future extension, researchers should develop heuristic algorithms to solve the problem with a higher number of roads or schedule for consecutive days. The model can be further extended by incorporating network formation to consider the influence of junctions, pollution control constraints, etc.

## Figures and Tables

**Figure A1:**
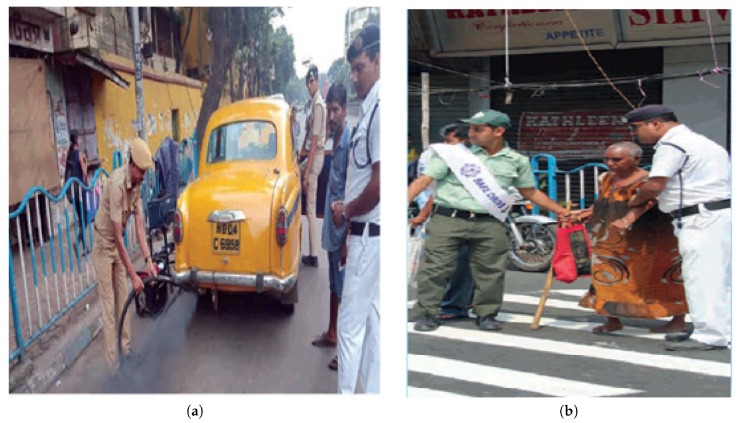
(**a**) Vehicle emission checking at roadside (**b**) Role of Civic-Volunteers (Kolkata Traffic Police Annual Review 2017, Pages 46 and 15).

## Appendix B. Detailed Numerical Results

We present the detailed numerical results in the following tables.

**Table A1:** Optimal allocation with increasing number of road segments using two-phase method.

Number of Roads		Obj 1	Obj 2	Obj 3	Obj 4	a	s	c	h	b	Total (a + s + c + h + v)	CPU Time (Sce.)
5	Obj1	**223,200**	0	158	449	81	0	19	140	18	258	95.15
	Obj2	430,000	**32**	116	1199	76	160	22	94	22	374	
	Obj3	333,700	12.81	**18**	999	86	135	19	0	18	258	
	Obj4	970,000	18.07	700	**2200**	100	160	200	300	400	1160	
	Phase2	**505,200**	**29.76**	**18**	**1541**	**61**	**160**	**183**	**0**	**18**	**422**	
6	Obj1	**257,600**	0	174	525	97	0	23	156	18	294	112.15
	Obj2	625,500	**29.33**	401	1475.50	97	160	25	218	183	683	
	Obj3	376,100	15.57	**18**	1129	98	155	17	0	18	288	
	Obj4	970,000	17.05	700	**2200**	100	160	200	300	400	1160	
	Phase2	**522,600**	**28.30**	**18**	**1574**	**93**	**160**	**162**	**0**	**18**	**433**	
7	Obj1	**294,300**	1	187	626	100	14	26	169	18	327	181.15
	Obj2	496,600	**32**	164	1332.50	98	160	28	145	19	450	
	Obj3	407,100	18.23	**41**	1198	100	160	22	23	18	323	
	Obj4	970,000	17.51	700	**2200**	100	160	200	300	400	1160	
	Phase2	**546,100**	**31.73**	**41**	**1615**	**100**	**160**	**161**	**23**	**18**	**462**	
8	Obj1	**326,800**	2.91	192	727	100	32	28	174	18	352	458.15
	Obj2	492,900	**31.87**	137	1350	100	160	41	117	20	438	
	Obj3	430,200	19.80	**64**	1242	100	160	29	46	18	353	
	Obj4	970,000	16.38	700	**2200**	100	160	200	300	400	1160	
	Phase2	**569,200**	**31.87**	**64**	**1659**	**100**	**160**	**168**	**46**	**18**	**492**	
9	Obj1	**365,300**	5.27	207	834	100	48	32	189	18	387	512.27
	Obj2	496,800	**31.47**	146	1352.50	100	160	40	119	27	446	
	Obj3	456,900	20.73	**95**	1288.00	100	160	34	77	18	389	
	Obj4	970,000	22.18	700	**2200.00**	100	160	200	300	400	1160	
	Phase2	**599,900**	**31.47**	**95**	**1717**	**100**	**160**	**177**	**77**	**18**	**532**	
10	Obj1	**405,200**	7.1905	224	943	100	64	36	206	18	424	514.27
	Obj2	522,400	**31.733**	176	1397.50	100	160	47	137	39	483	
	Obj3	487,000	18.833	**128**	1342	100	160	41	110	18	429	
	Obj4	970,000	20.029	700	**2200**	100	160	200	300	400	1160	
	Phase2	**619,000**	**30.45**	**128**	**1738**	**100**	**160**	**173**	**110**	**18**	**561**	
11	Obj1	**444,400**	7.89	240	1051	100	80	40	222	18	460	615.09
	Obj2	557,800	**30.08**	232	1442	100	160	43	194	38	535	
	Obj3	512,400	15.93	**160**	1383	100	160	44	142	18	464	
	Obj4	970,000	15.36	700	**2200**	100	160	200	300	400	1160	
	Phase2	**648,400**	**30.08**	**160**	**1791**	**100**	**160**	**180**	**142**	**28**	**610**	

**Table A2:** Optimal allocation with increasing number of road segments using weighted sum method.

Number of Roads	Obj1	Obj2	Obj3	Obj4	a	s	c	h	v	Total	CPU Time (Sec.)
5	223,200	0	158	449	81	0	19	140	18	258	13.41
6	257,600	0	172	526	99	0	22	154	18	293	19.81
7	294,300	1.6257	187	626	100	14	26	169	18	327	20.63
8	326,800	6.1667	192	727	100	32	28	174	18	352	42.97
9	365,300	8.9333	207	834	100	48	32	189	18	387	106.95
10	405,200	12.4	224	943	100	64	36	206	18	424	187.17
11	439,600	14.23	232	1045	100	80	40	218	14	452	366.21

**Table A3:** Sensitivity of optimal scheduling with increasing number of Sergeants.

Sergeants		Obj1	Obj2	Obj3	Obj4	a	s	c	h	v	Total Manpower (a + s + c + h + v)
60	Obj1	**294,300**	1.53	187	626	100	14	26	169	18	327
	Obj2	440,400	**24.00**	164	1143.50	99	120	31	143	21	414
	Obj3	380,100	13.78	**81**	1053	100	120	27	63	18	328
	Obj4	910,000	13.78	700	**2000**	100	120	200	300	400	1120
	Phase2	**533,100**	**24.00**	**81**	**1512**	**100**	**120**	**180**	**63**	**18**	**481**
80	Obj1	**294,300**	1.00	187	626	100	14	26	169	18	327
	Obj2	496,600	**32.00**	164	1332.50	98	160	28	145	19	450
	Obj3	407,100	18.23	**41**	1198	100	160	22	23	18	323
	Obj4	970,000	17.51	700	**2200**	100	160	200	300	400	1160
	Phase2	**546,100**	**31.73**	**41**	**1615**	**100**	**160**	**161**	**23**	**18**	**462**
100	Obj1	**294,300**	1.85	187	626	100	14	26	169	18	327
	Obj2	564,000	**40**	168	1549.50	100	200	31	145	23	499
	Obj3	419,900	19.73	**18**	1282	92	191	14	0	18	315
	Obj4	1,030,000	16.62	700	**2400**	100	200	200	300	400	1200
	Phase2	**581,000**	**38.97**	**18**	**1765**	**100**	**200**	**152**	**0**	**18**	**470**
120	Obj1	**294,300**	1.34	187	626	100	14	26	169	18	327
	Obj2	617,300	**48**	159	1739	100	240	30	139	20	529
	Obj3	423,500	24.14	**18**	1306	80	203	14	0	18	315
	Obj4	1,090,000	27.06	700	**2600**	100	240	200	300	400	1240
	Phase2	**605,000**	**46.97**	**18**	**1857**	**100**	**240**	**116**	**0**	**18**	**474**

**Table A4:** Sensitivity of optimal scheduling with respect to increasing cost of using volunteers (i = 7).

Volunteers		Obj1	Obj2	Obj3	Obj4	a	s	c	h	v	Total Manpower (a + s + c + h + v)
h-560	Obj1	**268,840**	0.80	187	626	100	14	26	169	18	327
v-400	Obj2	474,400	**32**	164	1332.50	98	160	28	145	19	450
	Obj3	402,080	18.23	**41**	1198	100	160	22	23	18	323
	Obj4	888,000	17.51	700	**2200**	100	160	200	300	400	1160
	Phase2	**522,080**	**31.73**	**41**	**1558**	**100**	**160**	**142**	**23**	**18**	**443**
h-630	Obj1	**281,570**	0.80	187	626.00	100	14	26	169	18	327
v-450	Obj2	485,500	**32.00**	164	1332.50	98	160	28	145	19	450
	Obj3	404,590	18.23	**41**	1198.00	100	160	22	23	18	323
	Obj4	929,000	17.51	700	**2200.00**	100	160	200	300	400	1160
	Phase2	**538,590**	**31.73**	**41**	**1600.00**	**100**	**160**	**156**	**23**	**18**	**457**
h-700	Obj1	**294,300**	1	187	626	100	14	26	169	18	327
v-500	Obj2	496,600	**32**	164	1332.5	98	160	28	145	19	450
	Obj3	407,100	18.233	**41**	1198	100	160	22	23	18	323
	Obj4	970,000	17.514	700	**2200**	100	160	200	300	400	1160
	Phase2	**546,100**	**31.73**	**41**	**1615**	**100**	**160**	**161**	**23**	**18**	**462**
h-770	Obj1	**307,030**	1.34	187	626.00	100	14	26	169	18	327
v-550	Obj2	507,700	**32.00**	164	1332.50	98	160	28	145	19	450
	Obj3	409,610	18.23	**41**	1198.00	100	160	22	23	18	323
	Obj4	1,011,000	17.51	700	**2200.00**	100	160	200	300	400	1160
	Phase2	**566,610**	**31.73**	**41**	**1669.00**	**100**	**160**	**179**	**23**	**18**	**480**
h-840	Obj1	**319,760**	1.34	187	626.00	100	14	26	169	18	327
v-600	Obj2	518,800	**32.00**	164	1332.50	98	160	28	145	19	450
	Obj3	412,120	18.23	**41**	1198.00	100	160	22	23	18	323
	Obj4	1,052,000	17.51	700	**2200.00**	100	160	200	300	400	1160
	Phase2	**577,120**	**31.73**	**41**	**1693.00**	**100**	**160**	**187**	**23**	**18**	**488**
